# Characterization of Patients With Refractory or Unexplained Chronic Cough Participating in a Phase 2 Clinical Trial of the P2X3-Receptor Antagonist Gefapixant

**DOI:** 10.1007/s00408-021-00437-7

**Published:** 2021-04-07

**Authors:** Alyn H. Morice, Surinder S. Birring, Jaclyn A. Smith, Lorcan P. McGarvey, Jonathan Schelfhout, Allison Martin Nguyen, Zhi Jin Xu, Wen-Chi Wu, David R. Muccino, Mandel R. Sher

**Affiliations:** 1 Respiratory Research Group, Hull York Medical School, Cottingham, UK; 2 Centre for Human & Applied Physiological Sciences, School of Basic & Medical Biosciences, Faculty of Life Sciences & Medicine, King’s College London, London, UK; 3 Division of Infection, Immunity, and Respiratory Medicine, University of Manchester, and Manchester University NHS Foundation Trust, Manchester, UK; 4 Wellcome-Wolfson Institute for Experimental Medicine, Queen’s University Belfast, Belfast, UK; 5 Merck & Co., Inc., Kenilworth, NJ USA; 6 Center for Cough, Largo, FL USA

**Keywords:** Persistent cough, Troublesome cough, Refractory cough, Idiopathic chronic cough, Cough hypersensitivity syndrome

## Abstract

**Purpose:**

This analysis assesses clinical characteristics of patients with refractory chronic cough (RCC) or unexplained chronic cough (UCC) enrolled in a phase 2 study to better understand this patient population.

**Methods:**

Patients with RCC/UCC lasting for ≥ 1 year and cough severity visual analog scale (VAS) score of > 40 mm at screening were eligible. Demographics, clinical characteristics, and medical history were collected at baseline. Cough-related measures included cough severity VAS, Cough Severity Diary (CSD), Leicester Cough Questionnaire (LCQ), and a structured cough-trigger questionnaire. Medication history included all medications 30 days before screening and chronic cough treatments within 1 year before screening. Data were summarized using descriptive statistics.

**Results:**

Patients (*N* = 253; female, 76%; mean age, 60 years) had severe (mean cough severity VAS, 57.5 mm) and long-lasting (median duration, 11 years) cough. The most burdensome self-reported aspects included psychological and social factors (LCQ) and cough frequency and intensity (CSD). Patient-reported triggers were consistent with cough hypersensitivity (e.g., 95% to 96% reported irritation or tickle in throat). Common reported comorbidities included gastroesophageal reflux disease (GERD; 56%), allergic rhinitis (47%), and asthma (30%); 12% of patients had been diagnosed with all 3 conditions. The most common prior medications included inhaled or oral steroids (21%), antihistamines (15%), and antacids (15%).

**Conclusion:**

Patients with RCC/UCC had severe, long-lasting, and burdensome cough with clinical features of cough hypersensitivity. Many patients had been diagnosed with GERD, allergic rhinitis, and asthma but had a persistent cough despite treatment of these conditions.

**Trial registration: **ClinicalTrials.gov, NCT02612610; registered November 20, 2015

## Introduction

Chronic cough (CC), defined as a cough lasting > 8 weeks, has a global prevalence of ~ 10% [1, 2]. Patients with CC can experience physical burden as a result of their cough and negative effects on their social lives and psychological well-being [3–5]. Chronic cough is often long lasting, persisting for several years and sometimes decades [5–8]. There is poor recognition that CC can be refractory to treatment of associated conditions or unexplainable; thus, patients with CC are frequently labeled as having other conditions, and such confusion often leads to multiple diagnoses being suspected [9, 10]. Diagnostic uncertainty may lead to repetitive medical consultations, fruitless investigations, and unnecessary treatment trials [4, 11, 12]. Patient burden is exacerbated by the current paucity of effective and safe treatment options for these patients, with no available pharmacologic treatments having approved indications for CC.

Intervention fidelity, defined as the “extent to which an intervention was delivered as conceived and planned to arrive at valid conclusions concerning its effectiveness in achieving the target outcomes,” is important for reliably identifying or excluding potential conditions associated with CC [13–15]. Diagnostic workup for CC includes the following steps: assessment of medical history to address factors that could contribute to cough (e.g., smoking; use of drugs that elicit cough, such as angiotensin-converting enzyme [ACE] inhibitors; environmental exposures); imaging and clinical assessments to identify red flags suggestive of life-threatening conditions; and differential diagnosis of potential comorbid conditions [1, 10]. Several medical conditions can be associated with CC, including gastroesophageal reflux disease (GERD), asthma, nonasthmatic eosinophilic bronchitis, and upper-airway cough syndrome (UACS). However, many patients with these conditions do not have CC, suggesting CC may be driven by distinct mechanisms [10]. Indeed, many patients with CC continue to cough despite optimal assessment and treatment of presumed associated conditions; these patients are often referred to as having refractory CC (RCC) [1, 10, 13]. Additionally, CC can be present in patients who do not have identifiable or treatable conditions associated with cough (defined here as unexplained CC [UCC]) [13]. Particularly for investigative clinical trials, it is important to confirm a patient has RCC/UCC to reliably evaluate novel treatments. The mechanisms underlying CC are under active investigation and are most likely heterogeneous [16]. However, many patients with CC share a common history of a cough triggered by low levels of thermal, mechanical, or chemical stimuli, including innocuous stimuli (allotussia), a sensation of itching or tickling in the throat (laryngeal paresthesia) accompanied by an urge to cough, and increased responsiveness to tussive stimuli (hypertussia) [17]. *Cough hypersensitivity syndrome* has been suggested as a useful clinical paradigm to describe these patients [13, 16–18].

An understanding of the typical profile of patients with CC is important to recognize the unique features of such patients. Epidemiologic studies have revealed typical features of patients with CC, including a preponderance of females who have never smoked [4, 11, 19–21]. The prevalence of CC peaks around middle age (i.e., in the fifth and sixth decades), though CC can occur in all age groups [22]. Patients with CC can be evaluated by using objective and subjective methods that measure different aspects of cough, including objective cough frequency, cough severity (e.g., with the Cough Severity Diary [CSD] or cough severity visual analog scale [VAS]), and cough-specific health-related quality of life (e.g., with the Leicester Cough Questionnaire [LCQ] or Cough-Specific Quality-of-Life Questionnaire) [23]. Although objective cough monitoring is used in clinical trials, patient-reported measures, such as a simple cough score (e.g., asking patients to score their cough severity on a scale from 1 to 10), are more widely used in clinical practice [10].

Most prior observational studies of CC involved single clinics and predominantly included patients with explained CC, with only a small proportion of RCC or UCC cases. The objective of this analysis was to characterize patients with RCC or UCC who were enrolled in a multicenter phase 2 study using a comprehensive, protocol-driven data collection approach to better define this patient population.

## Methods

### Study Design and Patient Population

This analysis assessed baseline demographics and characteristics of patients enrolled in a phase 2b, randomized, placebo-controlled trial of the P2X3-receptor antagonist gefapixant (ClinicalTrials.gov identifier, NCT02612610). Details regarding study design have been published [24]. For the current analysis, patients receiving any gefapixant dose or placebo were pooled into a single group for assessment of baseline characteristics.

Eligible patients were defined in the study protocol as having RCC or UCC based on American College of Chest Physicians (ACCP) and British Thoracic Society (BTS) guidelines. Patients were considered to have RCC if they had a clinical evaluation that identified at least one comorbid condition associated with CC but continued to cough despite receiving appropriate diagnostic workup and at least 2 months of therapy for the comorbid condition(s). Patients were defined as having UCC if there was no objective evidence of a comorbid condition associated with CC despite appropriate diagnostic workup per ACCP and BTS guidelines. Patients were required to have a minimum RCC or UCC duration of 1 year.

Additional inclusion criteria were age limits from 18 to 80 years and cough severity score of ≥ 40 mm on a 100-mm cough severity VAS at screening. Exclusion criteria included current smoking or recent smoking within 6 months of enrollment, a ratio of forced expiratory volume in 1 second to forced vital capacity of less than 60%, treatment initiation with an ACE inhibitor within 4 weeks of or during the study, use of opioids within 1 week of the study, or an upper or lower respiratory tract infection within 4 weeks of the study.

The study was performed in accordance with the International Council for Harmonisation-E6 Guideline for Good Clinical Practice and applicable federal regulations. All patients provided written informed consent before enrollment and all sites received approval from institutional review boards or independent ethics committees.

### Analysis Measures

Baseline cough-related characteristics included objective awake cough frequency and patient-reported outcomes. Objective cough frequency was measured at baseline in a 24-hours sound recording using a VitaloJAK (Vitalograph^®^; Vitalograph Ltd, Buckingham, United Kingdom) acoustic recording device as previously described [24, 25]. Patient-reported cough severity was scored from 0 to 100 using a 100-mm cough severity VAS (recall period: “past 24 hours”; 0 = not at all; 100 = extremely). Cough frequency, intensity, and disruption were assessed via the CSD, a 7-item questionnaire designed to be answered before bedtime (recall period: “today”); each item was measured on an 11-point numeric rating scale ranging from 0 (best) to 10 (worst). The CSD total score (range: 0 to 10) was calculated by averaging scores from all 7 items. Individual domain scores were calculated as the mean across items within each domain. The mean weekly CSD total score was calculated as the average CSD total score over the preceding week. Physical, psychological, and social burdens of cough were assessed using the LCQ, a 19-item health-related quality-of-life questionnaire (recall period: “last 2 weeks”). Each LCQ item was measured on a 7-point Likert scale, with lower numbers reflecting more severe cough effects. Individual scores for physical, psychological, and social domains were calculated as the average score across individual items within the domain; the LCQ total score (range: 3 to 21) was calculated as the sum of individual domain scores. The LCQ and CSD have been previously demonstrated to be reliable, responsive, and valid in patients with CC [26, 27]. Patients also completed a medical history questionnaire, developed for the purposes of this analysis, which comprised 21 items regarding various cough triggers. Responses regarding experience of individual triggers were categorized as either no (occurring “never” or “little of the time”) or yes (occurring “some of the time,” “a lot of the time,” “most of the time,” or “always”).

### Data Collection and Analysis

Medical history, including CC history, medication history (including over-the-counter medications administered 30 days before screening and CC treatments within 1 year before screening), and patient demographics were collected during screening. Comorbid conditions were identified on the basis of medical history in patient medical records. Medical history terms were coded using the MedDRA v19.0.

At the baseline visit, inclusion and exclusion criteria were confirmed, an updated medical history was obtained, concomitant medications were recorded, and baseline cough metrics (i.e., awake cough frequency, average of the daily CSD scores over the prior 7 days, cough severity VAS, and LCQ) were collected. Data were summarized using descriptive statistics.

## Results

### Patient Demographics and Characteristics

A total of 253 patients met eligibility criteria and were enrolled in the study. Most patients were female (76%), white (92%), and never smokers (70%). Median (range) age was 61 (22–79) years. On average, patients were overweight (mean body mass index, 27.7 kg/m^2^). All enrolled patients were from the United States or United Kingdom (Table 1).

**Table 1:** Baseline demographics and clinical characteristics [24]

Parameter	Patient population (*N* = 253)
Sex, *n* (%)	
Female	193 (76)
Male	60 (24)
Ethnicity, *n* (%)	
Hispanic or Latino	3 (1)
Not Hispanic or Latino	250 (99)
Race, *n* (%)	
American Indian or Alaskan native	2 (1)
Asian	3 (1)
Black or African American	12 (5)
White	234 (92)
Other	1 (< 1)
Multiple	1 (< 1)
Country, *n* (%)	
United Kingdom	88 (35)
United States	165 (65)
Age, years	
Mean (SD)	60 (10)
Median (range)	61 (22–79)
Smoking status, *n* (%)	
Never	177 (70)
Former	76 (30)
BMI, kg/m^2^	
Mean (SD)	27.7 (4.7)
FEV_1_/FVC, %	
Mean (SD)	81.7 (12.2)

*BMI* body mass index, *FEV*_*1*_ forced expiratory volume in 1 second, *FVC* forced vital capacity, *SD* standard deviation

### Baseline Cough Characteristics

Patients enrolled in this study had severe and long-lasting coughs (Table 2). Although eligibility criteria required patients to have a cough lasting for at least 1 year, the median duration of cough was much longer (11.0 years). Median awake cough frequency was 28.9 coughs per hour but ranged from less than 1 to more than 700 coughs per hour. Consistent with eligibility criteria, patients reported a mean baseline cough severity VAS score of 57.5 mm.

**Table 2:** Baseline cough characteristics

Parameter	Patient population (*N* = 253)
Cough duration, years	
Mean (SD)	14.2 (11.1)
Median (range)	11.0 (2.0, 56.0)
Awake cough frequency, coughs/hour^a^	
Mean (SD)	40.3 (55.8)
Geometric mean	26.9
Median (range)	28.9 (0.4, 734.0)
LCQ total score	
Mean (SD)	11.7 (3.0)
Median (range)	11.7 (4.2, 19.7)
Cough severity VAS score, mm^b^	
Mean (SD)	57.5 (22.3)
Median (range)	60.0 (7.0, 100.0)
Daily CSD total score^c^	
Mean (SD)	4.2 (1.9)
Median (range)	4.1 (0.8, 9.9)

*CSD* cough severity diary, *LCQ* Leicester Cough Questionnaire, *SD* standard deviation, *VAS* visual analog scale

^a^Includes patients who received at least one dose of placebo or gefapixant and provided one or more baseline and one or more postbaseline measures of awake cough frequency (*N* = 236)

^b^Baseline cough severity VAS scores were collected 1 to 14 days after collection of cough severity VAS scores to determine trial eligibility during screening

^c^*N* = 246 for baseline CSD

**Fig. 1:**
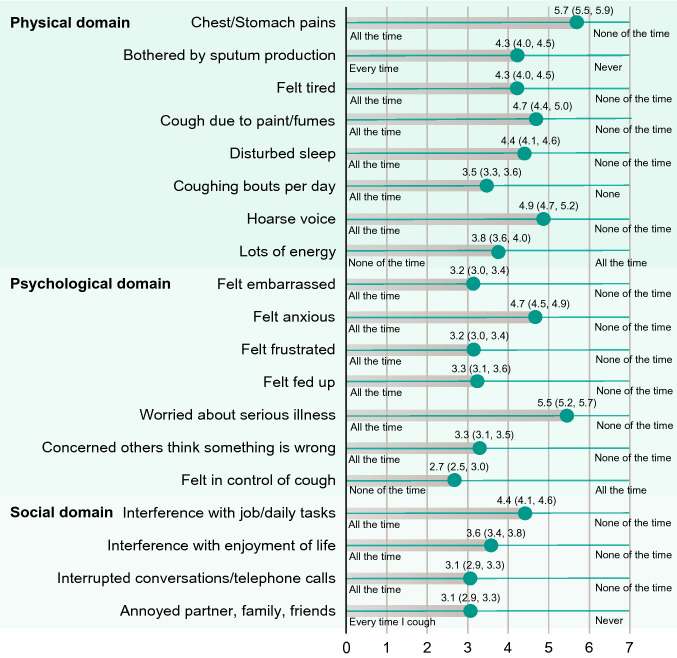
Individual items in the Leicester Cough Questionnaire reported by patients at baseline. Data expressed in mean (95% confidence interval)

The mean total LCQ score at baseline was 11.7, with mean scores of 4.4, 3.7, and 3.5 for the physical, psychological, and social domains, respectively. Baseline total LCQ scores ranged from 4.2 to 19.7. Individual LCQ items with the highest reported burden (i.e., lowest scores) included psychological factors (did not feel in control of cough, embarrassment, frustration) and social factors (annoying to partner, family, and friends; interrupted conversations). Items in the physical domain were typically reported as less burdensome compared with the psychological and social domains, with chest/stomach pains and hoarse voice among the items with lowest reported overall burden (Fig. 1).

**Fig. 2:**
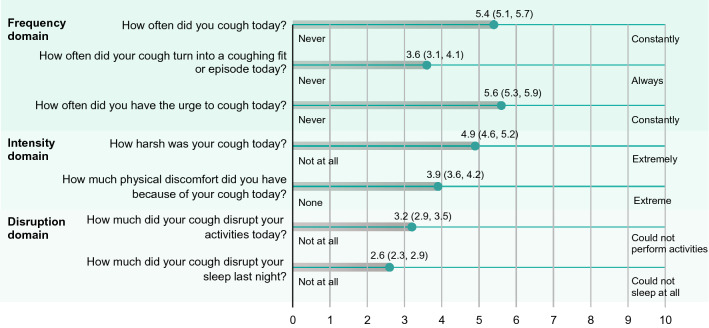
Individual items in the Cough Severity Diary reported by patients at baseline. Data expressed in mean (95% confidence interval)

The mean weekly CSD total score at baseline was 4.2, with mean scores of 4.9, 4.4, and 2.9 for the frequency, intensity, and disruption domains. Baseline mean weekly CSD total scores ranged from 0.8 to 9.9. Items that were rated as most burdensome were in the frequency (frequency of urge to cough, cough frequency) and intensity (harshness of cough) domains (Fig. 2). Items related to disruption (disruption of activities or sleep) were rated as least burdensome.

Each cough trigger included in the baseline 21-item questionnaire was self-reported to elicit cough at least some of the time by more than 30% of patients (Table 3). The most common triggers of cough were irritation in the throat (88%) and a tickle in the throat (85%). Only 4% to 5% of patients reported that irritation or tickling in the throat never elicited their cough. The most common external triggers were poor air quality (74%) and change in air temperature (71%). The least commonly reported triggers were crying (32%), swallowing (35%), and certain foods (44%). Nearly two-thirds of patients (65%) reported there were no specific triggers for their cough and 90% reported their coughing was often unpredictable.

**Table 3:** Reported triggers of cough (*n* = 252)

Item	Yes (*some of the time* to *always*) *n* (%)	No (*little of the time* to *never*) *n* (%)
My cough is unpredictable	228 (90)	24 (10)
An irritation in my throat triggers my cough	223 (88)	29 (12)
A tickle in my throat triggers my cough	213 (85)	38 (15)
Poor air quality triggers my cough	187 (74)	65 (26)
A change in air temperature triggers my cough	180 (71)	72 (29)
I cough because I need to clear my throat	180 (71)	72 (29)
Deep breathing makes me cough	165 (65)	87 (35)
There are no specific triggers for my cough	163 (65)	89 (35)
Exertion or exercise makes me cough	163 (65)	89 (35)
A dry throat triggers my cough	158 (63)	94 (37)
Laughing triggers my cough	155 (62)	97 (38)
I cough after meals	148 (59)	104 (41)
Lying flat makes me cough	148 (59)	104 (41)
Changes in the weather trigger my cough	144 (57)	108 (43)
A high pollen count triggers my cough	142 (56)	110 (44)
Breathlessness causes me to cough	142 (56)	110 (44)
My cough is triggered by certain smells and odors	140 (56)	112 (44)
An irritation in my chest has triggered my cough	128 (51)	123 (49)
My cough is triggered by certain foods	111 (44)	141 (56)
Swallowing triggers my cough	89 (35)	163 (65)
Crying triggers my cough	80 (32)	172 (68)

One patient did not complete the trigger questionnaire at baseline

### Medical History and Comorbidities

**Fig. 3:**
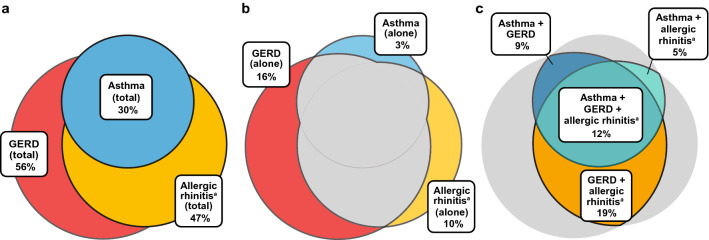
Most common medical conditions associated with chronic cough in the patient population. **a** Percentage of patients with a medical history of GERD, asthma, or allergic rhinitis; **b** percentage of patients with a single diagnosis of GERD, asthma, or allergic rhinitis; and **c** percentage of patients with dual or all 3 diagnoses of GERD, asthma, or allergic rhinitis. GERD, gastroesophageal reflux disease. ^a^Diagnostic codes in *allergic rhinitis* category include *seasonal rhinitis*, *seasonal allergic rhinitis*, *seasonal allergies rhinitis*, *perennial seasonal allergic rhinitis (dust mite)*, and *seasonal allergic rhinitis (trees weeds grass)*

Common comorbidities associated with CC were prevalent in the patient population; 56%, 30%, and 47% of patients had a diagnosis of GERD, asthma, and allergic rhinitis, respectively, recorded in their medical history (Fig. 3a). Overall, 29% of patients had a single diagnosis of one of these conditions (Fig. 3b), 33% had dual diagnoses, and 12% had diagnoses of all 3 conditions (Fig. 3c).

The most common classes of prior or comorbid conditions were identified using patient medical records (Table 4). Several individual-condition medical history items were consistent with complications or comorbidities related to cough, including headache (15%), hiatus hernia (13%), stress urinary incontinence (5%), and bronchitis (4%).

**Table 4:** Commonly reported medical conditions by body system class (> 20%)

Parameter	Patient population (*n* = 252) *n* (%)
Respiratory, thoracic, and mediastinal disorders (including cough)	252 (100)
Surgical and medical procedures	181 (72)
Gastrointestinal disorders (e.g., gastroesophageal reflux disease)	174 (69)
Musculoskeletal and connective tissue disorders	139 (55)
Nervous system disorders	121 (48)
Infections and infestations	112 (44)
Social circumstances (e.g., menopause, postmenopause)	106 (42)
Metabolism and nutrition disorders	99 (39)
Vascular disorders	97 (38)
Psychiatric disorders	92 (37)
Immune system disorders	84 (33)
Skin and subcutaneous tissue disorders	56 (22)

One patient did not report medical history

The most common medication classes were consistent with cough treatments and associated comorbid conditions (Table 5). The most common individual medications used before enrollment that were presumed to be cough related included gabapentin (11%), benzonatate (9%), omeprazole (8%), prednisone (7%), bromhexine (6%), azelastine hydrochloride (6%), and guaifenesin (5%).

**Table 5:** Most common prior therapies (*n* = 252)

Medication class	*n* (%)
Steroids (inhaled or oral)	54 (21)
Antihistamines	39 (15)
Stomach acid reducers^a^	39 (15)
Bronchodilators	36 (14)
Benzonatate	23 (9)
Opioids	21 (8)

Includes all medications, including over-the-counter medications, over the past 30 days that were recorded, in addition to all chronic cough treatments over the past year at screening

One patient did not report medical history

^a^Includes proton pump inhibitors, antacids, and H_2_-receptor antagonists

## Conclusion

This analysis assessed characteristics of patients with RCC/UCC enrolled in a large phase 2 study. Patients were typically female, middle aged, never smokers, and overweight. They presented with long-lasting, severe, and burdensome cough, as evidenced by a median cough duration of more than a decade, high awake cough frequency, and severe cough based on patient-reported outcomes (i.e., CSD, cough severity VAS). Patients also had an impaired cough-specific health-related quality of life, with the highest self-reported burden being related to social and psychological impairment. Patients reported a wide range of cough triggers, including those implicated in cough hypersensitivity syndrome (e.g., 95% to 96% of patients reported at least some throat tickle or irritation). Patients frequently had 1 or more diagnoses of conditions thought to be associated with CC.

The clinical profile of patients with RCC/UCC enrolled in this phase 2 study is generally consistent with that of several previous epidemiologic and observational CC studies, although they included patients with explained CC and did not select by cough severity [4, 20–22]. Particularly, a retrospective study of 10,032 patients with CC who attended specialist cough clinics and did not have significant radiologic abnormalities found that patients were predominantly female (66%), had a mean age of 55 years, and had a common age of presentation in the sixth decade, consistent with demographics observed in the current analysis [22]. However, the proportion of female patients in the current study (76%) was notably greater than the proportion of females (57%) in a previous large population-based study that included 554 individuals with CC [21]. This analysis expands upon previous studies by providing additional, prospective characterization of the RCC/UCC patient population using both objective and subjective measures. Although the clinical profile identified in this and previous studies may help identify these patients in a clinical setting, a definitive diagnosis of RCC/UCC should be made using established guidelines [1, 10]. Of note, the current study was not designed to compare characteristics between patients diagnosed with RCC vs UCC, and future research comparing these populations may be warranted to assess whether these diagnostic labels reflect distinct patient populations.

Strengths of the analysis include a large sample size of a well-defined group of patients with RCC or UCC. The use of a clinical study protocol with defined eligibility criteria is more likely to include a consistent diagnosis of RCC/UCC than that reported in observational studies. Additionally, the study protocol facilitated collection of information that may not be readily available in observational studies, including medical history, prior treatments and medications, awake cough frequency, and patient-reported outcomes.

There were limitations to this analysis. First, the selected clinical study population of patients with RCC/UCC with a cough severity VAS score of > 40 mm may not represent patients with less severe RCC/UCC. Second, medical histories, including comorbid conditions, were identified from diagnoses recorded in patients’ medical records; although clinical diagnoses (such as GERD and UACS) may have been accurately captured, medical conditions that relied on patient reports could have been underestimated because specific questions were not asked. For example, the prevalence of stress urinary incontinence observed in this study (5%) was much lower than the prevalence of stress urinary incontinence reported in a study that used targeted questions to estimate the prevalence of incontinence in women with CC (63%) [28]. Third, prior medications and CC treatments were only recorded over the past 30 days and 1 year, respectively; thus, only current or recent treatments were captured. Of note, patients enrolled in this study had experienced CC for many years, so collection of a comprehensive medication history would be challenging. Fourth, the medical history questionnaire used to collect data regarding cough triggers did not have a defined recall period and was not intended to capture potential variation in cough-trigger prevalence over time. Finally, all enrolled patients were from the United States or United Kingdom; it is therefore unclear how these results may generalize to patients from other geographic regions.

This analysis suggests that patients with RCC/UCC have a common clinical profile that is generally consistent with previous studies of patients with CC.

## Data Availability

The data sharing policy of Merck Sharp & Dohme Corp., a subsidiary of Merck & Co., Inc., Kenilworth, NJ, USA, including restrictions, is available at http://engagezone.msd.com/ds_documentation.php. Requests for access to the clinical study data can be submitted through the EngageZone site or via email to dataaccess@merck.com.
